# Outcomes of the Paul Glaucoma Implant in an Ambispective Multicenter Study: 12-Month Follow-Up

**DOI:** 10.3390/biomedicines14061230

**Published:** 2026-05-29

**Authors:** Celina Logioco, Anahí Lupinacci, Ignacio Lischinsky, Mariel A. Ytques, Gabriel Bercovich, María A. Moussalli, Agustina De Gainza, Mario O. Roux, Nicolás Levaggi, Ana Sanseau, Karina B. Giannone, Arturo Burchakchi, Leila Galetto, Natanael Serrano, Eimi Olivares Sefair, Guillermo Roux, Rodrigo M. Torres

**Affiliations:** 1 Centro de Ojos Quilmes, Quilmes B1878KDF, Argentina; leilagaletto@gmail.com; 2 Department of Ophthalmology, Hospital Universitario Austral, Pilar B1629, Argentina; anahilupi@gmail.com (A.L.); serranonatanael@gmail.com (N.S.); 3 Centro Oftalmológico Lischinsky, Tucumán T4000, Argentina; nachomdm@gmail.com; 4 Consulta Privada, Cipolletti R8324, Argentina; mytques@gmail.com; 5 Clínica Oftalmológica Bercovich, Rosario S2000, Argentina; gabrielbercovich@gmail.com; 6 Hospital Italiano de Buenos Aires, Ciudad de Buenos Aires C1199, Argentina; mamoussalli@hotmail.com (M.A.M.); arturo.burchakchi@hospitalitaliano.org.ar (A.B.); 7 Consultores Oftalmológicos, Ciudad de Buenos Aires C1018, Argentina; adegainza1@gmail.com; 8 Clínica Roux, San Juan J5402, Argentina; mosvaldoroux@yahoo.com.ar (M.O.R.); eimiolivares@gmail.com (E.O.S.); guillermo_roux@hotmail.com (G.R.); 9 Hospital Lagleyze, Ciudad de Buenos Aires C1416, Argentina; nicolevaggi@gmail.com; 10 Gonella Oftalmólogos, Ciudad de Buenos Aires C1122, Argentina; asanseauoftalmo@gmail.com; 11 Consulta Privada, CIMEF, Junín de Los Andes Q8371, Argentina; karinagiannone@gmail.com; 12 R.O.M.A.T. Creator Center, Colonia Avellaneda E3107, Argentina

**Keywords:** glaucoma surgery, glaucoma drainage devices, Paul glaucoma implant, intraocular pressure, glaucoma treatment, ambispective multicenter study

## Abstract

**Objective:** The aim of this study was to evaluate the safety and efficacy of the Paul Glaucoma Implant (PGI) in a multicenter Argentine cohort with 12 months of follow-up. **Methods:** This ambispective multicenter study included patients who underwent PGI implantation between November 2022 and July 2024 by glaucoma specialists across Argentina, with a minimum follow-up of 12 months. Primary outcomes were intraocular pressure (IOP) reduction and success rates, defined as complete success (≥20% IOP reduction with IOP ≥6 and ≤21 mmHg without medications), qualified success (same criteria with ≥1 medication), and failure based on predefined efficacy and safety criteria, including additional glaucoma surgery, device removal, or clinically significant hypotony. Secondary outcomes included changes in medication use, best-corrected visual acuity (BCVA), and postoperative complications. **Results:** Sixty-six eyes were included in the overall analysis. Mean IOP decreased from 31.2 ± 9.1 mmHg preoperatively to 12.8 ± 4.7 mmHg at 12 months (*p* < 0.01). Medications were reduced from 3.5 ± 0.8 to 1.3 ± 1.2 (*p* < 0.01). Among the 65 eyes with evaluable 12-month follow-up, 50 eyes (76.9%) achieved complete success, 14 (21.5%) qualified success, and 1 (1.5%) failure. Complications were ocular hypertension (25%), tube or plate exposure (10%), choroidal detachment (7%), and hypotony (5%). BCVA remained stable in 28 eyes (43.8%), improved in 15 (23.4%), and worsened in 21 (32.8%). **Conclusions:** The PGI achieved significant and sustained IOP reduction with marked medication decrease at 12 months. Most complications were mild or moderate. These findings support the PGI as an effective and safe non-valved implant for refractory glaucoma in Latin American populations.

## 1. Introduction

Glaucoma remains one of the leading causes of irreversible blindness worldwide, and refractory glaucoma represents a particularly challenging subgroup because it often requires surgical strategies beyond conventional filtering procedures. Glaucoma drainage devices (GDDs) have become a central component in the surgical management of advanced and refractory glaucoma, particularly in eyes with high risk of failure after conventional filtering procedures [1,2,3,4,5]. Contemporary surgical algorithms emphasize individualized selection of surgical techniques according to glaucoma subtype, prior interventions, and ocular comorbidities, with tube shunt implantation frequently recommended in complex scenarios such as neovascular, uveitic, or pseudoexfoliative glaucoma and in eyes with previous surgical failure [1,4,5]. Recent systematic reviews and meta-analyses comparing established devices, including Ahmed and Baerveldt implants, highlight the continued evolution of GDD technology and the need to balance efficacy with safety, particularly regarding hypotony and long-term intraocular pressure (IOP) control [6,7].

Among refractory glaucoma subtypes, neovascular glaucoma (NVG) remains one of the most challenging to manage surgically due to its aggressive clinical course, inflammatory milieu, and high risk of postoperative complications [8,9]. A recent systematic review and meta-analysis of randomized trials demonstrated substantial heterogeneity in surgical approaches and outcomes for NVG, underscoring the absence of consensus regarding optimal management and the importance of real-world evidence to guide clinical decision-making [5]. Similarly, pseudoexfoliative and uveitic glaucomas present distinct anatomical and inflammatory characteristics that may influence surgical success and complication profiles following drainage device implantation [4,10].

The Paul Glaucoma Implant (PGI) has recently emerged as a novel non-valved aqueous shunt designed to optimize the balance between effective IOP reduction and the risk of postoperative hypotony [11,12]. Its design incorporates a large endplate surface area and a reduced internal tube diameter compared with conventional non-valved devices, aiming to enhance long-term flow control while minimizing early postoperative overfiltration [12]. Early clinical series and recent systematic reviews have reported encouraging efficacy and safety outcomes with the PGI, positioning it as a promising alternative within the spectrum of contemporary glaucoma drainage devices [13,14,15,16,17,18,19,20,21,22]. Nevertheless, variability in reporting standards, outcome definitions, and complication classification across glaucoma surgical studies remains a significant limitation when comparing results between cohorts and devices [23,24].

Regional and population-specific factors may further influence surgical outcomes and device performance. Differences in glaucoma epidemiology, access to subspecialty care, and surgical practice patterns across Latin America may affect both indications and postoperative evolution of GDD implantation [2,25]. Preferred practice recommendations from the Latin American Glaucoma Society emphasize the need for locally generated evidence to complement data derived predominantly from European, Asian, and North American cohorts and to guide context-specific surgical decision-making [2]. In this context, multicenter real-world evaluations are particularly relevant for implantable surgical devices, as they allow assessment of reproducibility across surgeons and clinical environments while capturing the heterogeneity inherent to routine clinical practice.

Against this background, the present study reports the first national multicenter Argentine real-world study with the Paul Glaucoma Implant, evaluating its safety and efficacy over 12 months in a heterogeneous cohort of patients with refractory glaucoma. The study aims to provide region-specific real-world evidence regarding intraocular pressure control, reduction in medication burden, and postoperative complications associated with PGI implantation in routine clinical practice.

## 2. Materials and Methods

### 2.1. Study Design

A multicenter ambispective study was conducted, including all patients who underwent implantation of the PGI by glaucoma specialists from different provinces of Argentina between November 2022 and July 2024. Data collection was standardized across centers after ethics approval on 31 July 2024. For eyes that had already completed the 12-month follow-up at the time of study initiation, data were collected retrospectively from the medical records. For eyes that were still under follow-up at that time, preoperative, intraoperative, and prior postoperative data were collected retrospectively, whereas subsequent follow-up data were recorded prospectively during scheduled postoperative visits. Thus, the study combined fully retrospective cases with cases contributing both retrospective and prospective follow-up data. Only eyes with a minimum follow-up of 12 months were analyzed. The study protocol was reviewed and approved by the Ethics Committee of the Argentine Council of Ophthalmology (CAO-04/24), and all procedures adhered to the tenets of the Declaration of Helsinki.

### 2.2. Clinical Parameters

Demographic data, glaucoma subtype, and lens status were recorded. Glaucoma subtypes were categorized as primary open-angle glaucoma (POAG), pseudoexfoliative glaucoma (PXG), neovascular glaucoma (NVG), uveitic glaucoma, congenital glaucoma, post-vitrectomy glaucoma, or “other,” which included eyes with trauma, angle-closure glaucoma, or mixed etiologies. IOP and the number of glaucoma medications were measured preoperatively and at postoperative visits: day 1, week 1, month 1, month 3, month 6, and month 12.

### 2.3. Efficacy Outcomes

Efficacy was evaluated according to predefined outcome domains aligned with World Glaucoma Association and European Glaucoma Society principles for glaucoma surgical trials. The primary efficacy outcome was cumulative surgical success at 12 months. Complete success was defined as ≥20% IOP reduction from baseline with IOP ≥ 6 and ≤21 mmHg without hypotensive medications. Qualified success was defined as the same IOP criteria but requiring ≥1 medication. Failure was defined as <20% IOP reduction from baseline, IOP > 21 mmHg, the need for additional glaucoma surgery, device removal, loss of light perception, or persistent hypotony (IOP ≤ 5 mmHg on two consecutive visits after 3 months), particularly when associated with clinical consequences or requiring intervention, in accordance with contemporary glaucoma surgical reporting principles [26,27].

### 2.4. Safety Outcomes

Safety was assessed by best-corrected visual acuity (BCVA), with a loss of up to two lines considered acceptable. Numerical hypotony was defined as IOP ≤ 5 mmHg, whereas clinical hypotony was defined as low IOP associated with clinically significant sequelae, such as shallow anterior chamber, choroidal detachment, hypotony maculopathy, visual loss, or the need for surgical intervention. Complications were classified according to timing as intraoperative, early postoperative (≤3 months), or late postoperative (>3 months), and graded as mild (self-limiting), moderate (requiring medical but not surgical treatment), or severe (requiring surgical reintervention). Reoperations excluded minor slit-lamp procedures such as laser suture lysis, laser iridoplasty, intraluminal stent removal, subconjunctival needling, or anterior chamber viscoelastic injection.

### 2.5. Surgical Technique

Paul Glaucoma Implant surgery was performed by experienced glaucoma surgeons using a broadly shared operative framework, although some technical variations were accepted according to surgeon preference, ocular characteristics, and the real-world multicenter nature of the study. In general, a superior conjunctival–Tenon’s pocket was created in the superotemporal or superonasal quadrant, depending on the eye and surgical indication. Conjunctiva and Tenon’s capsule were carefully dissected from the sclera, and the adjacent rectus muscles were isolated with strabismus hooks to ensure adequate exposure and mobilization of the implantation site. In many cases, mitomycin C (0.3 mg/mL) was applied for 3 min, although its use was not uniform across all surgeons.

Before implantation, the tube was primed posteriorly and an intraluminal Prolene 6-0 stent was inserted to modulate early postoperative flow. The implant plate was then positioned beneath the conjunctiva and under the adjacent rectus muscles. The tube was inserted into the anterior chamber or ciliary sulcus according to lens status and surgeon judgment, using a scleral tunnel created with a 25-gauge needle. After insertion, the tube was secured to the sclera with 10-0 nylon sutures to minimize movement. Tube coverage was achieved either with a scleral patch graft or with a partial-thickness scleral flap fashioned in a trabeculectomy-like manner, depending on surgeon preference. Conjunctival closure was performed with 10-0 Vicryl or 10-0 nylon.

Because of the ambispective design, surgery had already been completed in all cases at the time the multicenter study was organized. Therefore, although the overall operative principles were consistent across centers, some inter-surgeon variations in antimetabolite use, tube coverage, and flow-restriction maneuvers were present and were accepted as part of the real-world character of the study.

### 2.6. Statistical Analysis

Descriptive statistics were used to summarize demographic and clinical data. No formal sample size calculation was performed, as this was conceived as a real-world multicenter series including all eligible consecutive cases during the study period. The normality of continuous variables was assessed with the Kolmogorov–Smirnov test. Continuous variables are expressed as mean ± standard deviation (SD) and range, and categorical variables as counts and percentages. Comparisons of mean IOP and number of hypotensive medications across follow-up visits (baseline, months 1, 3, 6, and 12) were performed using one-way repeated-measures analysis of variance (ANOVA), with Bonferroni-adjusted post hoc comparisons. A *p*-value < 0.05 was considered statistically significant. Success rates (complete, qualified, and failure) were calculated according to predefined efficacy criteria, and proportions were descriptively compared between subgroups such as glaucoma subtype and lens status. Changes in best-corrected visual acuity were summarized as improvement, stability, or worsening (≤2 or >2 lines). Kaplan–Meier survival analysis was performed to estimate the cumulative probability of complete and qualified surgical success over the 12-month follow-up period. All statistical analyses were performed using XL Miner (Google Sheets add-on, Mountain View, CA, USA), and all figures were generated using Python (version 3.11; Python Software Foundation, Wilmington, DE, USA) with the Matplotlib library (version 3.13).

## 3. Results

Of 69 eyes initially enrolled, 3 were excluded because they were lost to follow-up before completing 12 months. Therefore, 66 eyes were included in the overall analysis. One eye developed refractory clinical hypotony requiring explantation of the device during the first postoperative week and was classified as a surgical failure. As this eye did not complete the 12-month follow-up, analyses requiring 12-month IOP and medication data were based on the remaining 65 eyes (31 men, 34 women; mean age 54.6 ± 14.8 years, range 19–82) operated by 13 surgeons. Lens status was aphakic in 2 eyes (3.1%), phakic in 30 (46.1%), and pseudophakic in 33 (50.8%). Glaucoma types included neovascular (22, 32.3%), uveitic (10), POAG (9), pseudoexfoliative (6), congenital (3), post-vitrectomy (1), and unspecified (14). The implant was placed superotemporally in 61 eyes, superonasally in 3, and inferotemporally in 1; antimetabolites were used in 39 eyes (MMC 37, 5-FU 2).

**Figure 1:**
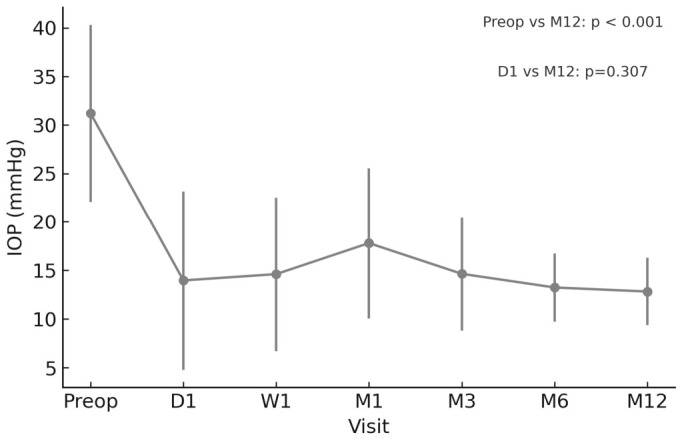
Mean intraocular pressure (IOP) at each follow-up visit up to 12 months after surgery with the Paul Glaucoma Implant, among eyes with evaluable 12-month follow-up data. *p*-values correspond to comparisons between baseline and month 12, and between day 1 and month 12.

**Figure 2:**
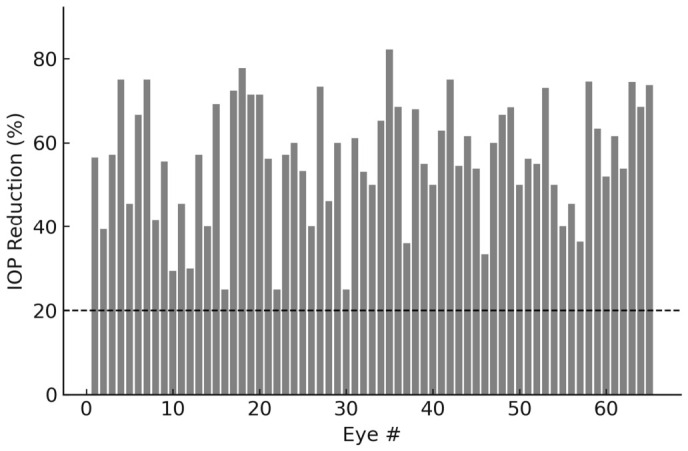
Individual IOP reduction (%) from baseline to 12 months among eyes with evaluable 12-month follow-up data (n = 65). The dashed line marks the 20% success threshold.

**Figure 3:**
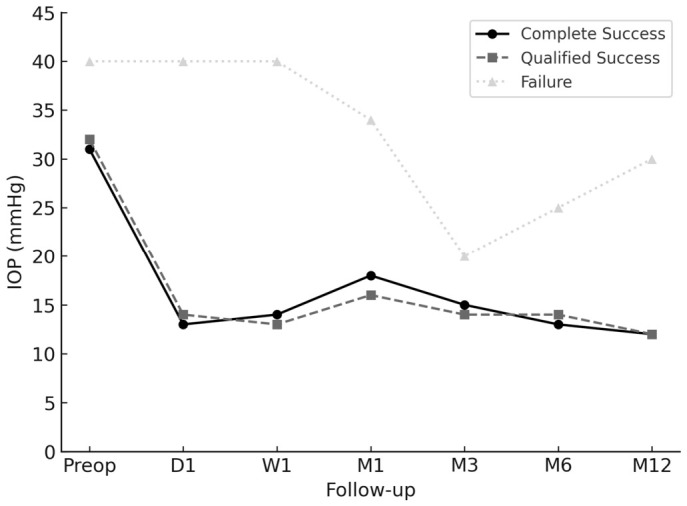
Mean intraocular pressure over time by outcome group (complete success, qualified success, failure) among eyes with evaluable 12-month follow-up. D; Day; W: week; M: Month.

Mean IOP decreased from 31.2 ± 9.1 mmHg at baseline to 12.8 ± 4.7 mmHg at month 12 (*p* < 0.01), with medications reduced from 3.5 ± 0.8 to 1.3 ± 1.2 (*p* < 0.01) (Table 1, Figure 1 and Figure 2). Among the 65 eyes with 12-month evaluable data, complete success was achieved in 50 eyes (76.9%), qualified success in 14 (21.5%), and failure in 1 (1.5%, pseudophakic uveitic eye, final IOP 30 mmHg on 4 medications) (Figure 3, Table 2). The eye that required explantation because of refractory clinical hypotony during the first postoperative week was classified separately as an early surgical failure.

Complications (Table 3) included ocular hypertension (15 eyes, 25%), tube/plate exposure (6, 10%), choroidal detachment (4, 7%), and hypotony (3, 5%). Less common events were subconjunctival hemorrhage (5%), cataract progression (3%), corneal graft failure (3%), hyphema (3%), corneal dellen (3%), vitreous hemorrhage (2%), and macular edema (2%). One case required explantation, and several required conjunctival/scleral patching or graft surgery.

At 12 months, BCVA was stable in 28 eyes (43.8%), improved in 15 (23.4%), worsened by ≤2 lines in 8 (12.5%), and worsened by >2 lines in 13 (20.3%). In the complete success group, 36 eyes remained stable or within ≤2 lines, 2 improved, and 2 worsened >2 lines; in the qualified success group, 9 were stable, 3 improved, and 2 worsened >2 lines. The failure identified at 12 months showed no visual change. The eye that underwent early explantation because of refractory clinical hypotony was analyzed separately as an early surgical failure.

**Figure 4:**
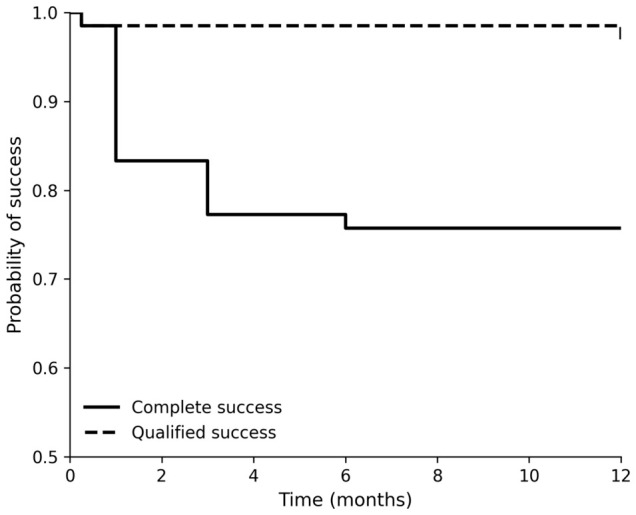
Kaplan–Meier survival curves showing the cumulative probability of maintaining complete success (solid line) and qualified success (dashed line) after Paul Glaucoma Implant implantation over 12 months.

Kaplan–Meier survival analysis demonstrated the cumulative probability of complete and qualified surgical success over the 12-month follow-up period (Figure 4). An early decline in both curves reflects one case of refractory clinical hypotony requiring explantation during the first postoperative week. The complete success curve showed additional stepwise declines over time corresponding to the introduction of adjunctive hypotensive medication.

## 4. Discussion

This multicenter ambispective study provides robust real-world evidence on the performance of the PGI in a heterogeneous cohort of refractory glaucomas and represents, to our knowledge, the first national multicenter experience reported from Latin America. The present findings confirm that the PGI achieves substantial and sustained IOP reduction with a favorable safety profile at 12 months, supporting its role as an effective non-valved drainage device in complex clinical scenarios. Importantly, the consistency of outcomes across multiple surgeons and centers suggests that the device performs reproducibly in routine clinical practice and not only in highly specialized single-center environments.

The magnitude and trajectory of IOP reduction observed in our cohort closely parallel those reported in PGI series from Europe and Asia, reinforcing the external validity of the device’s performance across diverse populations and healthcare systems [11,12,13,14,15,16,17,18,19]. Complete and qualified success rates in the present study fall within the upper range of previously reported outcomes. A recent systematic review encompassing 946 eyes undergoing PGI implantation reported complete success rates ranging from 38% to 75% and qualified success rates exceeding 85%, with consistent and sustained IOP reduction across studies [20,21,22]. In this context, our complete success rate of 76.9% and qualified success rate of 21.5% align with the higher spectrum of published outcomes, further supporting the effectiveness of the device in real-world clinical practice. The relatively high complete success rate observed in our series may reflect both the flow-restrictive design of the PGI and structured perioperative management strategies across participating centers. These findings collectively support the PGI as a reliable option for sustained IOP control in refractory glaucoma.

A distinctive feature of this multicenter cohort is the high proportion of secondary glaucomas, including neovascular and uveitic cases, which are traditionally associated with more aggressive disease course and less predictable surgical outcomes [5,8,9,10]. Despite this complexity, the PGI demonstrated substantial pressure reduction and acceptable complication rates across subgroups. These results align with prior reports in uveitic and pseudoexfoliative glaucoma and suggest that the PGI may maintain efficacy even in highly inflammatory or ischemic ocular environments [4,17,18]. Given the known variability in outcomes for neovascular glaucoma and the absence of clear consensus regarding optimal surgical strategies, the present findings contribute meaningful real-world evidence supporting the use of non-valved drainage devices in these challenging contexts.

Subgroup patterns in our cohort also deserve comment. Complete success was more frequent in pseudoexfoliative and primary open-angle glaucoma, whereas uveitic glaucoma showed a greater proportion of qualified rather than complete success, suggesting that postoperative pressure control was achievable in many of these eyes, although often with the need for adjunctive medication. Despite its recognized surgical complexity and poorer prognosis, neovascular glaucoma still accounted for a substantial proportion of successful cases in this series. Although these findings should be interpreted cautiously because of the limited sample size within each subgroup, they suggest that the PGI may provide clinically meaningful IOP control across different glaucoma subtypes, including secondary glaucomas traditionally considered at higher surgical risk.

From a device-design perspective, the PGI incorporates structural characteristics intended to optimize aqueous outflow resistance while minimizing the risk of early postoperative hypotony. The combination of a reduced internal tube diameter and a relatively large endplate surface area may facilitate controlled filtration and stable long-term pressure reduction. This design rationale aligns with broader trends in glaucoma drainage device development aimed at balancing safety and sustained efficacy. Meta-analyses of established implants, such as Ahmed and Baerveldt devices, continue to highlight the trade-off between hypotony risk and long-term pressure control in glaucoma surgery, reinforcing the need for continued innovation in device design [6,7]. Other contemporary drainage devices developed in Latin America, such as the Susanna glaucoma drainage device introduced in Brazil, have incorporated specific structural modifications intended to improve flow control and surgical handling [28]. Early clinical reports have demonstrated encouraging safety and efficacy outcomes at one year of follow-up, suggesting that regional innovation in glaucoma drainage technology continues to evolve [29]. However, published data remain limited and, to date, there are no multicenter or real-world reports evaluating this device in Argentina. In this context, the present multicenter evaluation of the PGI contributes important region-specific evidence and highlights the growing relevance of locally generated data for assessing the performance of emerging glaucoma implants in diverse clinical settings.

The complication profile observed in this series was consistent with previously reported PGI cohorts and with expectations for non-valved drainage devices in complex glaucomas. Rates of hypotony and tube or plate exposure fell within published ranges and were generally manageable with medical or surgical interventions [19,20,21,22]. In earlier single-center experiences, Studsgaard et al. reported hypotony in approximately 6% of cases, whereas longer-term follow-up from other cohorts has documented rates as high as 35% at three years, highlighting the influence of follow-up duration and patient characteristics on reported outcomes [16,19]. Similarly, Richardson et al. reported a hypotony rate of 2% in a predominantly uveitic cohort and an 8% failure rate at one year [18]. In our series, one eye met failure criteria at the 12-month evaluation and one additional eye required early explantation because of refractory clinical hypotony. These variations across studies underscore the heterogeneity of patient populations and surgical contexts and reinforce the importance of region-specific, real-world data. Early postoperative ocular hypertension, frequently observed during the first postoperative months, likely reflects the well-recognized hypertensive phase associated with endplate encapsulation and wound healing responses, a phenomenon common to many non-valved implants. Variability in complication rates across studies further emphasizes the need for standardized definitions and reporting frameworks in glaucoma surgical research [23,24].

One of the most relevant contributions of this study is the demonstration of consistent outcomes across multiple surgeons and clinical centers. Reproducibility of surgical results is a critical but often underreported dimension in the evaluation of implantable devices. The homogeneous performance observed in this multicenter setting suggests that the PGI can achieve reliable outcomes beyond single-expert environments, supporting its broader applicability in routine practice. Multicenter real-world evaluations are particularly valuable for surgical devices, as they capture variability in patient characteristics and surgical approaches while providing a more comprehensive assessment of effectiveness and safety.

The generation of region-specific evidence also contributes to addressing persistent geographic disparities in glaucoma research and surgical innovation. Most published data on glaucoma drainage devices originate from Europe, North America, and parts of Asia, with comparatively limited representation from Latin America and other regions [2,25]. By providing multicenter data from a Latin American cohort, this study helps expand the global evidence base and supports more inclusive and context-sensitive clinical decision-making in glaucoma surgery.

Strengths of this study include its multicenter design and the consistency of outcomes observed across different surgeons and clinical settings, supporting the reproducibility of PGI implantation in routine practice. The inclusion of a heterogeneous cohort with a high proportion of secondary glaucomas further enhances the external validity of the findings and reflects real-world clinical conditions.

Several limitations should nevertheless be acknowledged. The ambispective design and absence of a randomized comparator group preclude direct comparison with other glaucoma drainage devices. The relatively short follow-up period does not allow definitive conclusions regarding long-term device survival or late complications. In addition, variability in perioperative management across centers may have influenced outcomes, although this heterogeneity also mirrors real-world practice. Ethnic background was not collected in a standardized manner across participating centers and therefore could not be analyzed. Likewise, standardized structural and functional assessments, including visual field and OCT parameters, were not uniformly available across all sites and were therefore not included as predefined study outcomes. Future prospective and comparative studies with longer follow-up will be essential to better define predictors of long-term success and to further establish the role of the PGI within the contemporary surgical armamentarium for refractory glaucoma.

## 5. Conclusions

In summary, the Paul Glaucoma Implant demonstrated favorable safety and efficacy outcomes in this first national multicenter Argentine series, with a high proportion of eyes achieving complete or qualified success at 12 months. Significant and sustained reductions in intraocular pressure and medication burden were observed across a heterogeneous cohort that included complex glaucoma subtypes. Visual acuity remained stable or improved in most cases, and postoperative complications were generally manageable and consistent with those reported for non-valved drainage devices. These findings support the PGI as a reproducible and effective surgical option for refractory glaucoma in real-world clinical practice, and suggest that it may be considered in centers managing complex glaucomas, provided that appropriate postoperative monitoring is available. In addition, this study contributes region-specific evidence to inform surgical decision-making in Latin America. Further prospective and comparative studies with longer follow-up are warranted to better define its long-term performance and optimal indications.

## Figures and Tables

**Table 1:** Intraocular pressure and number of hypotensive medications in patients undergoing Paul Glaucoma Implant surgery.

*p*	Month 12	Month 6	Month 3	Month 1	Week 1	Day 1	Preop	
<0.01	12.8 ± 4.7	12.8 ± 6.3	14.2 ± 5.9	17.8 ± 7.7	14.6 ± 7.9	13.9 ± 9.2	31.2 ± 9.1	IOP
	(7–30)	(5–20)	(6–35)	(5–42)	(3–38)	(4–44)	(17–56)	
<0.01	1.3 ± 1.2 (0–4)	1.4 ± 1.0 (0–3)	1.6 ± 1.0 (0–4)	1.4 ± 1.4 (0–4)	1.4 ± 1.4 (0–4)	1.3 ± 1.4 (0–4)	3.5 ± 0.8 (1–4)	n° of drugs

IOP: intraocular pressure; n°: number. Month 12 values were calculated from the 65 eyes with evaluable 12-month follow-up. One additional eye, included in the overall analysis, underwent early explantation during the first postoperative week because of refractory clinical hypotony and was classified separately as an early surgical failure. Values are expressed as mean ± standard deviation (range). IOP: intraocular pressure; n°: number.

**Table 2:** Distribution of glaucoma subtypes and lens status in eyes achieving complete or qualified success at 12 months.

Qualified Success (n)	Complete Success (n)	Lens Status	Glaucoma Subtype
0	1	Phakic	Congenital
0	1	Pseudophakic	
1	0	Aphakic	
1	5	Phakic	Primary Open Angle (POAG)
0	3	Pseudophakic	
0	1	Phakic	Pseudoexfoliative (PXG)
0	5	Pseudophakic	
0	1	Aphakic	Neovascular
2	10	Phakic	
2	7	Pseudophakic	
1	4	Phakic	Other
2	6	Pseudophakic	
1	0	Pseudophakic	Post-vitrectomy
3	1	Phakic	Uveitic
1	5	Pseudophakic	

**Table 3:** Postoperative complications observed after Paul Glaucoma Implant surgery.

Outcome	Management	Time of Occurrence	Eyes (n, %)	Complication
Controlled in most cases	Medication adjustment or Prolene removal	M1–M3	15 (25%)	Ocular hypertension
Resolved in 2, 1 failure	Conservative in 2, explant in 1	D1–W1	3 (5%)	Hypotony
Variable, one case required device removal one month after surgery	Conjunctival/scleral patch, some explanted	M3–M12	6 (10%)	Tube/plate exposure
Resolved without sequelae	Steroids, cycloplegics, systemic therapy	M1–M6	4 (7%)	Choroidal detachment
Resolved	Observation or AC washout	W1–M1	2 (3%)	Hyphema
Gradual improvement	Observation	D1–M1	1 (2%)	Vitreous hemorrhage
Resolved	Clip suture or conservative	W1	3 (5%)	Subconjunctival hemorrhage
Good visual recovery	Phacoemulsification	M6–M12	2 (3%)	Cataract progression
Repeat transplant performed	Repeat keratoplasty	M6–M12	2 (3%)	Corneal graft failure
Vision-threatening, some required device removal	Conservative or device removal	M1–M12	2 (3%)	Corneal dellen
VA improved after treatment	Intravitreal anti-VEGF	M6	1 (2%)	Macular edema
Variable	Case-specific	Variable	5 (8%)	Other (allergic reaction, conjunctival wound dehiscence, etc.)

D: day; W: week; M: month.

## Data Availability

The datasets generated and analyzed during the current study are not publicly available due to patient privacy considerations and the diversity of institutional and regulatory requirements across participating centers; however, anonymized data may be made available from the corresponding author upon reasonable academic request.

## Abbreviations

The following abbreviations are used in this manuscript:

D	Day
IOP	Intraocular pressure
M	Month
n°	number
W	Week

## References

[1] Gedde S.J., Herndon L.W. (2025). Glaucoma Surgery: From the Tried and True to the Novel and New. Ophthalmol. Glaucoma.

[2] Justiniano M.J., Mura J.J., Giampani Junior J., Silva M.J.L., Fong G.B. (2026). Surgical procedures in glaucoma: A preferred practice pattern report by the Latin American Glaucoma Society. Arq. Bras. Oftalmol..

[3] Fellman R.L., Mattox C., Singh K., Flowers B., Francis B.A., Robin A.L., Butler M.R., Shah M.M., Giaconi J.A., Sheybani A. (2020). American Glaucoma Society Position Paper: Microinvasive Glaucoma Surgery. Ophthalmol. Glaucoma.

[4] Gillmann K., Meduri E., Niegowski L.J., Mermoud A. (2021). Surgical Management of Pseudoexfoliative Glaucoma: A Review of Current Clinical Considerations and Surgical Outcomes. J. Glaucoma.

[5] Ramji S., Nagi G., Ansari A.S., Kailani O. (2023). A systematic review and meta-analysis of randomised controlled trials in the management of neovascular glaucoma: Absence of consensus and variability in practice. Graefes Arch. Clin. Exp. Ophthalmol..

[6] Matarazzo F., Passaro M.L., Rinaldi M., Afflitto G.G., Aiello F., Avolio F.C., Aurilia A., Strianese D., Nucci C., Costagliola C. (2025). Ahmed and baerveldt in glaucoma surgery: What is the safest choice?—A systematic review and meta-analysis. Graefes Arch. Clin. Exp. Ophthalmol..

[7] Sebhat A.M., Sarhan H.A., Alfatih M., Hidad A.G., Aref A.A., Sayed M.S., Elhusseiny A.M. (2026). Outcomes of the Ahmed clearpath glaucoma drainage device in glaucoma: A systematic review. Graefes Arch. Clin. Exp. Ophthalmol..

[8] Rao A., Padhi T.R., Khan S.M. (2025). Clinical Challenges with Neovascular Glaucoma-Patient Tailored Strategies and Outcomes. Niger. Med. J..

[9] Gerçeker Demircan S., Şanal Doğan A. (2025). Neovascular glaucoma: Comprehensive evaluation of etiology, treatment modalities, and visual prognosis. BMC Ophthalmol..

[10] Fujita A., Elze T., Zhao Y., Lorch A.C., Miller J.W., Friedman D.S., Zebardast N., IRIS® Registry Analytic Center Consortium (2026). Comparison of Glaucoma Surgery Incidence and Outcomes in Pseudoexfoliation and Primary Open-Angle Glaucoma: IRIS^®^ Registry Analysis. Ophthalmology.

[11] Koh V., Chew P., Triolo G., Lim K.S., Barton K. (2020). PAUL Glaucoma Implant Study Group. Treatment Outcomes Using the PAUL Glaucoma Implant to Control Intraocular Pressure in Eyes with Refractory Glaucoma. Ophthalmol. Glaucoma.

[12] Vallabh N.A., Mason F., Yu J.T.S., Yau K., Fenerty C.H., Mercieca K., Spencer A.F., Au L. (2022). Surgical technique, perioperative management and early outcome data of the PAUL^®^ glaucoma drainage device. Eye.

[13] José P., Barão R.C., Teixeira F.J., Marques R.E., Peschiera R., Barata A., Abegão Pinto L. (2022). One-Year Efficacy and Safety of the PAUL Glaucoma Implant Using a Standardized Surgical Protocol. J. Glaucoma.

[14] Tan M.C.J., Choy H.Y.C., Koh Teck Chang V., Aquino M.C., Sng C.C.A., Lim D.K.A., Loon S.C., Chew Tec Kuan P. (2022). Two-Year Outcomes of the Paul Glaucoma Implant for Treatment of Glaucoma. J. Glaucoma.

[15] Weber C., Hundertmark S., Liegl R., Jauch A.S., Stasik I., Holz F.G., Mercieca K. (2023). Clinical outcomes of the PAUL^®^ glaucoma implant: One-year results. Clin. Exp. Ophthalmol..

[16] Tan M.C.J., Ong C.W., Aquino M.C., Lun K.W., Sng C.C.A., Lim D.K.A., Loon S.C., Koh V.T.C., Chew P.T.K. (2024). Three-Year Outcomes of the Paul Glaucoma Implant for Treatment of Glaucoma. J. Glaucoma.

[17] Olgun A., Karapapak M. (2024). Assessing the Efficacy of the PAUL Glaucoma Implant in Pseudoexfoliative Glaucoma. Beyoglu Eye J..

[18] Richardson J., Tacea F., Yu J., Yau K., Fenerty C., Au L. (2025). The PAUL Glaucoma Implant in the management of uveitic glaucoma-3-year follow-up. Eye.

[19] Studsgaard A., Nielsen S.E., Telinius N. (2025). One tube for all: 1-year outcomes after transition to Paul glaucoma implant at a tertiary centre. Acta Ophthalmol..

[20] Carlà M.M., Gambini G., Boselli F., Hu L., Perugini A.M., Crincoli E., Catania F., De Luca L., Rizzo S. (2025). The Paul Glaucoma Implant: A systematic review of safety, efficacy, and emerging applications. Graefes Arch. Clin. Exp. Ophthalmol..

[21] Tan N., Summers S., Alryalat S.A., Patnaik J.L., Lazcano-Gomez G.S., Seibold L.K., Kahook M.Y. (2025). A Systematic Review of the PAUL Glaucoma Implant. Clin. Ophthalmol..

[22] Chan K.E., Chan N.S., Liu M., Aquino M.C., Koh V.T.C., Lun K.W., Lim D.K.A., Loon S.C., Chew P.T.K., Tan M.C.J. (2025). Clinical Efficacy and Safety of the PAUL Glaucoma Implant: A Systematic Review and Meta-Analysis. Clin. Exp. Ophthalmol..

[23] Sii S., Barton K., Pasquale L.R., Yamamoto T., King A.J., Azuara-Blanco A. (2018). Reporting Harm in Glaucoma Surgical Trials: Systematic Review and a Consensus-Derived New Classification System. Am. J. Ophthalmol..

[24] Servillo A., Cutolo C.A., Viganò C., Forte P., Manocchio R., Gazzard G., Rossetti L., Iester M., Oddone F., Virgili G. (2026). Adherence of glaucoma intervention studies to World Glaucoma Association guidelines. Acta Ophthalmol..

[25] Bondok M., Dewidar O., Al-Ani A., Selvakumar R., Ing E., Ramke J., El-Hadad C., Damji K.F., Li T., Welch V. (2025). Inequities in glaucoma research: An analysis of Cochrane systematic reviews and randomized trials. J. Clin. Epidemiol..

[26] Weinreb R.N., Ramulu P., Topouzis F., Park K.H., Mansouri K., Lerner F. (2019). Glaucoma Surgery: 11th Consensus Report of the World Glaucoma Association.

[27] Abegao Pinto L., Sunaric Mégevand G., Stalmans I., Azuara-Blanco A., Bron A., Garcia Feijoo J., Garway Heath T., Grehn F., King A., Kirwan J. (2023). European Glaucoma Society—A guide on surgical innovation for glaucoma. Br. J. Ophthalmol..

[28] Prata T.S., Paranhos A. (2017). Clinical Implications of Specific Features of the New Susanna Glaucoma Drainage Device. J. Glaucoma.

[29] Susanna F.N., Susanna B.N., Susanna C.N., Nicolela M.T., Susanna R. (2021). Efficacy and Safety of the Susanna Glaucoma Drainage Device After 1 Year of Follow-up. J. Glaucoma.

